# Photocatalytic degradation of methyl orange at different pH values by NaYF_4_:Yb^3+^,Tm^3+^@TiO_2_ microrod composite photocatalysts under NIR excitation

**DOI:** 10.1039/d6ra00088f

**Published:** 2026-05-29

**Authors:** Yanna Tang, Jia Zeng

**Affiliations:** a Ningbo Polytechnic University Ningbo Zhejiang 315800 China 2015076@nbpt.edu.cn +86-13736135197

## Abstract

In this work, NaYF_4_:Yb^3+^,Tm^3+^@TiO_2_ microrod composite photocatalysts were fabricated *via* a hydrothermal method combined with a two-step wet-chemical coating strategy. Scanning electron microscopy (SEM) and transmission electron microscopy (TEM) characterization demonstrated that the NaYF_4_:Yb^3+^,Tm^3+^ microrods (∼1.2 µm in length and ∼0.4 µm in width) were uniformly coated with a porous anatase TiO_2_ shell. For the optimal sample S3, the thickness of the TiO_2_ shell was approximately 10 nm, with the size of a single TiO_2_ nanoparticle being about 6 nm. Under excitation with a 980 nm near-infrared (NIR) laser (spot size: 2 cm × 2 cm, total power: 28 W, and effective power density: 7 W cm^−2^), the TiO_2_ shell could efficiently absorb the ultraviolet upconversion emission from the NaYF_4_:Yb^3+^,Tm^3+^ core, and fluorescence kinetic measurements confirmed that fluorescence resonance energy transfer (FRET) occurs between the core and the shell. Compared with hydrothermally treated NaYF_4_:Yb^3+^,Tm^3+^, the fluorescence lifetime of the Tm^3+ 1^I_6_ excited state (at 291 nm emission) in the composite decreased from 128 µs to 100 µs, with a reduction of approximately 21%; in contrast, and the lifetime of the Tm^3+ 1^G_4_ excited state (at 474 nm emission) increased from 413 µs to 456 µs, increasing by about 10%. Photocatalytic degradation experiments with methyl orange (MO, 10 mg L^−1^, 50 mL) as the target pollutant revealed that the pH value of the solution exerted a significant influence on the degradation efficiency. Under the experimental conditions of 1 mg mL^−1^ catalyst concentration and 70 W cm^−2^ excitation power density (catalyst dosage: 50 mg, room temperature, magnetic stirring at 500 rpm, and 30 min dark adsorption equilibrium), the composite exhibited the optimal photocatalytic performance in an acidic system at pH 2, where the decolorization efficiency of MO was significantly higher than that in the neutral system (pH 6.5) and alkaline system (pH 11). Moreover, the MO decolorization rate decreased sequentially with an increase in the pH value of the solution. This study confirms that the FRET process between NaYF_4_:Yb^3+^,Tm^3+^ and TiO_2_ is a crucial mechanism for enhancing the NIR-driven photocatalytic activity of composite photocatalysts.

## Abstract 2

NaYF_4_:Yb^3+^,Tm^3+^@TiO_2_ microrod composite photocatalysts were fabricated *via* a hydrothermal method combined with a two-step wet-chemical coating strategy. The core role of FRET in enhancing their NIR photocatalysis was revealed.

## Graphical abstract

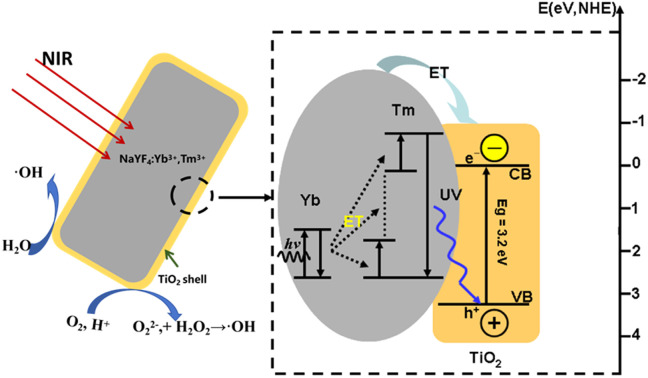

## 1. Introduction

As a highly promising green technology, photocatalytic CO_2_ reduction can convert carbon dioxide into organic fuels, which can alleviate the greenhouse effect and realize the recycling of carbon resources.^1,2^ In the field of environmental protection, the antibacterial and disinfection capabilities of photocatalysis can effectively inactivate microorganisms, such as bacteria and viruses, thus ensuring environmental safety.^3^ In biomedicine, photodynamic therapy based on photocatalytic principles provides novel strategies for cancer treatment.

Currently, photocatalytic technology still faces several critical challenges.^4,5^ Most traditional photocatalysts can only absorb in the ultraviolet region of sunlight, leading to a low utilization rate of visible light, which accounts for a larger proportion of the solar spectrum.^6,7^ Additionally, the photogenerated electron–hole pairs are prone to rapid recombination, which reduces the quantum efficiency and restricts the improvement of photocatalytic performance.^8^ Meanwhile, the difficulty in separating and recovering photocatalysts from reaction systems also hinders their large-scale industrial application.

Rare-earth-doped upconversion nanoparticles can convert near-infrared light into ultraviolet/visible emission, offering a promising way to utilize NIR light.^9^ In our previous studies, we first achieved NIR-responsive photocatalysis using YF_3_:Yb^3+^,Tm^3+^/TiO_2_ core/shell nanoparticles and further developed NaYF_4_:Yb,Tm@TiO_2_ with high NIR-driven activity and an elucidated energy transfer mechanism.^10,11^To address the above issues, in this work, NaYF_4_:Yb^3+^,Tm^3+^@TiO_2_ microrod composite photocatalysts were prepared *via* a hydrothermal method. We systematically investigated the NIR-driven photocatalytic properties of the as-prepared microrod composites for methyl orange degradation at different solution pH values, and clarified the intrinsic mechanism of the enhanced photocatalytic activity from the perspective of energy transfer.

## 2. Preparation of NaYF_4_:Yb^3+^,Tm^3+^@TiO_2_ microcomposites

### 2.1. reagents and instruments

Reagents used in the experiment: Yttrium nitrate (Y(NO_3_)_3_, 99.999%), ytterbium nitrate (Yb(NO_3_)_3_, 99.999%), thulium nitrate (Tm(NO_3_)_3_, 99.999%), ethylenediaminetetraacetic acid (EDTA, analytical grade), sodium fluoride (NaF), tetrabutyl titanate (TBOT, analytical grade), absolute ethanol and deionized water.

Experimental instruments and equipment: electronic balance, high-speed centrifuge, bench-top ultrasonic cleaner, magnetic stirrer and stainless-steel reaction kettle with a polytetrafluoroethylene lining.

### 2.2 Preparation of NaYF_4_:Yb^3+^,Tm^3+^ microrods

Ethylenediaminetetraacetic acid (EDTA) was employed as a surface modifier and complexing agent to synthesize hexagonal-phase NaYF_4_:Yb^3+^,Tm^3+^ microrods *via* a hydrothermal method. The molar ratio of the rare earth (RE) ions Y^3+^:Yb^3+^:Tm^3+^ was precisely controlled at 79 : 20 : 1, corresponding to the molar percentages of 79.0 mol% Y^3+^, 20.0 mol% Yb^3+^ and 1.0 mol% Tm^3+^ in the RE ion component. The actual molar ratios of Y^3+^, Yb^3+^ and Tm^3+^ in the prepared microrods were verified by inductively coupled plasma atomic emission spectroscopy (ICP-AES). The test results showed that the actual molar percentages of the three RE ions were consistent with the preset feeding ratio (deviation <1%), confirming the accurate control of the RE ion doping ratio in the synthesis process.

The specific experimental procedure is as follows: 1 mmol of mixed rare earth nitrates (prepared strictly according to the above 79 : 20 : 1 molar ratio) and 1 mmol of EDTA were added to a specific volume of deionized water, and the mixture was stirred magnetically for about 30 minutes until a stable white complex was formed. Then, an aqueous solution containing 12 mmol of NaF was added dropwise to the above complex solution under continuous stirring, and the stirring was maintained for 1 hour to ensure uniform mixing. The resulting precursor solution was transferred to a 40 mL closed autoclave with a polytetrafluoroethylene lining, and the hydrothermal reaction was conducted at 180 °C for 12 hours. After the reaction was completed, the autoclave was cooled naturally to room temperature. The obtained white precipitate was washed several times with absolute ethanol and deionized water to remove residual reactants, and finally dried in a vacuum drying oven at 80 °C for 12 hours to obtain the pure NaYF_4_:Yb^3+^,Tm^3+^ microrod powder.

### 2.3 Preparation of NaYF_4_:Yb^3+^,Tm^3+^@TiO_2_ core–shell composites

The concentrations of the as-prepared NaYF_4_:Yb^3+^,Tm^3+^ microcrystals and tetrabutyl titanate (TBOT) in the reaction precursor solution were fixed at 2 mg mL^−1^ and 3 µL mL^−1^, respectively. Titania-coated NaYF_4_:Yb^3+^,Tm^3+^ core–shell particles were fabricated by regulating the hydrolysis and condensation reactions of titanium alkoxide in ethanol. Here, titanium *n*-butoxide (Ti(OBu)_4_) was employed as the Ti source because its hydrolysis rate is approximately 150 times slower than that of tetraethyl titanate (Ti(OEt)_4_), which is conducive to the formation of a uniform and compact TiO_2_ shell layer.

A typical coating procedure was carried out as follows: NaYF_4_:Yb^3+^,Tm^3+^ nanoparticles were uniformly dispersed in ethanol containing a trace amount of Lutensol ON50 aqueous solution under ultrasonication. Subsequently, titanium alkoxide dispersed in anhydrous ethanol was added dropwise to the above seed particle dispersion at a controlled rate under rigorous magnetic stirring, and the reaction was allowed to proceed at room temperature for 20 h to form the TiO_2_ shell precursor. To obtain crystalline TiO_2_ shells, the above precursor solution was transferred to a 40 mL autoclave, sealed, and subjected to hydrothermal treatment at 160 °C for 6 h. The resulting NaYF_4_:Yb^3+^,Tm^3+^@TiO_2_ core–shell product was collected by centrifugation, washed with distilled water and anhydrous ethanol several times to remove the unreacted titanium alkoxide and by-products, and then dried in an air-drying oven at 80 °C to a constant weight.

### 2.4 Preparation of core–shell microrods with different TiO_2_ shell thicknesses

For the controllable synthesis of NaYF_4_:Yb^3+^,Tm^3+^@TiO_2_ core–shell composites with different TiO_2_ shell thicknesses, the concentrations of NaYF_4_:Yb^3+^,Tm^3+^ microcrystals and tetraethyl titanate (Ti(OEt)_4_) in the reaction precursor solution were adjusted, and the corresponding feeding amounts are listed in Table 1.

**Table 1:** Concentrations of NaYF_4_:Yb^3+^,Tm^3+^ and Ti(OEt)_4_ in the reaction precursor solution

Sample	NaYF_4_:Yb^3+^,Tm^3+^ (mg mL^−1^)	Ti(OEt)_4_ (µL mL^−1^)
S1	10	3
S2	5	3
S3	2	3
S4	2	6

## 3. Characterization and analysis

The purity and phase structure of the resulting products were analyzed on a Rigaku model Ru-200b X-ray powder diffractometer (XRD) using nickel-filtered Cu Kα radiation (*λ* = 1.5406 Å) in the 2*θ* range from 20° to 70°. Their size and morphology were characterized using transmission electron microscopy (TEM, JEM 2010F) at an accelerating voltage of 100 kV. The samples for TEM investigations were prepared by first dispersing the particles in ethanol under ultrasonication and then depositing a drop of the suspension onto a copper TEM grid coated with a holey carbon film. Photoluminescence spectra were recorded at room temperature on a Hitachi F-4500 fluorescence spectrophotometer (1.0 nm for slit width and 400 V for PMT voltage) with excitation from a 980 nm diode laser.

### 3.1 Structure and morphology

#### 3.1.1 XRD

The structure and morphology of the samples were analyzed through X-ray diffraction (XRD), scanning electron microscopy (SEM), transmission electron microscopy (TEM), and high-resolution transmission electron microscopy (HRTEM). Figures show the characterization results of the NaYF_4_:Yb^3+^,Tm^3+^ microcrystals and NaYF_4_:Yb^3+^,Tm^3+^@TiO_2_ composites. All measurements were performed at room temperature.

**Fig. 1:**
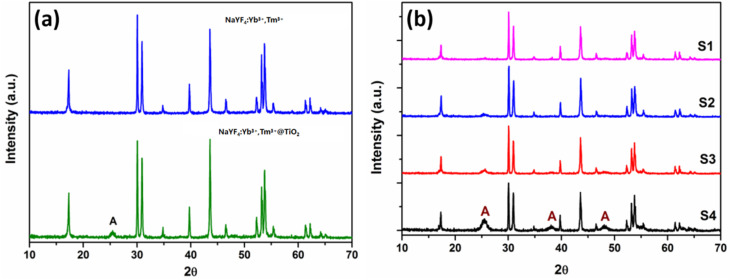
(a) XRD patterns of the pristine NaYF_4_:Yb^3+^,Tm^3+^ microrods and NaYF_4_:Yb^3+^,Tm^3+^@TiO_2_ core–shell composites, with the standard reference pattern of hexagonal NaYF_4_ (JCPDS No. 16-0334) and anatase TiO_2_ (JCPDS No. 21-1272) overlaid for phase identification. (b) XRD patterns of the NaYF_4_:Yb^3+^,Tm^3+^@TiO_2_ composite samples S1–S4, where the characteristic peak marked A corresponds to the (101) crystal plane of anatase TiO_2_ (JCPDS No. 21-1272), and the hexagonal NaYF_4_ (JCPDS No. 16-0334) standard reference pattern is overlaid as the reference for the main phase.

The crystal phase structure of the samples was analyzed using a Rigaku Ru-200b powder X-ray diffractometer (*λ* = 1.5406 Å) in the 2*θ* scanning range of 10° to 70°. Fig. 1(a) presents the XRD patterns of NaYF_4_:Yb^3+^,Tm^3+^ before and after TiO_2_ coating, with the standard reference patterns of hexagonal NaYF_4_ (JCPDS No. 16-0334) and anatase TiO_2_ (JCPDS No. 21-1272) overlaid for direct phase comparison. All the diffraction peaks of the pristine NaYF_4_:Yb^3+^,Tm^3+^ microrods perfectly match the standard pattern of hexagonal NaYF_4_ (JCPDS No. 16-0334), without any impurity diffraction peaks, indicating the high phase purity of the as-synthesized NaYF_4_:Yb^3+^,Tm^3+^. The sharp diffraction peaks with narrow full width at half maximum (FWHM) further confirm the excellent crystallinity of the hexagonal NaYF_4_ phase. For the NaYF_4_:Yb^3+^,Tm^3+^@TiO_2_ core–shell composites, a weak but distinct diffraction peak is observed at 2*θ* = 25.4°, which is matches well with the characteristic (101) diffraction peak of anatase TiO_2_ (JCPDS No. 21-1272). The relatively low intensity and broad FWHM of this TiO_2_ peak suggest the small particle size of the anatase TiO_2_ shell. Notably, the position and intensity of the main diffraction peaks of hexagonal NaYF_4_ in the composites remain unchanged, confirming that the hydrothermal coating and crystallization process of TiO_2_ do not alter the crystal phase structure of the NaYF_4_:Yb^3+^,Tm^3+^ core.

Fig. 1(b) shows the XRD patterns of composite samples S1–S4 with the hexagonal NaYF_4_ standard pattern (JCPDS No. 16-0334) overlaid. The results further verify that all the composite samples retain the pure hexagonal phase of NaYF_4_:Yb^3+^,Tm^3+^, and neither the TiO_2_ coating process nor the variation in TiO_2_ content induces a phase transformation of the NaYF_4_ core. With an increase in the titanium source concentration in the precursor solution, the intensity of the anatase TiO_2_ characteristic peak (2*θ* = 25.4°, JCPDS No. 21-1272, marked as A) gradually increases, which is consistent with the increase in TiO_2_ content in the core–shell composites.

#### 3.1.2 SEM and TEM

**Fig. 2:**
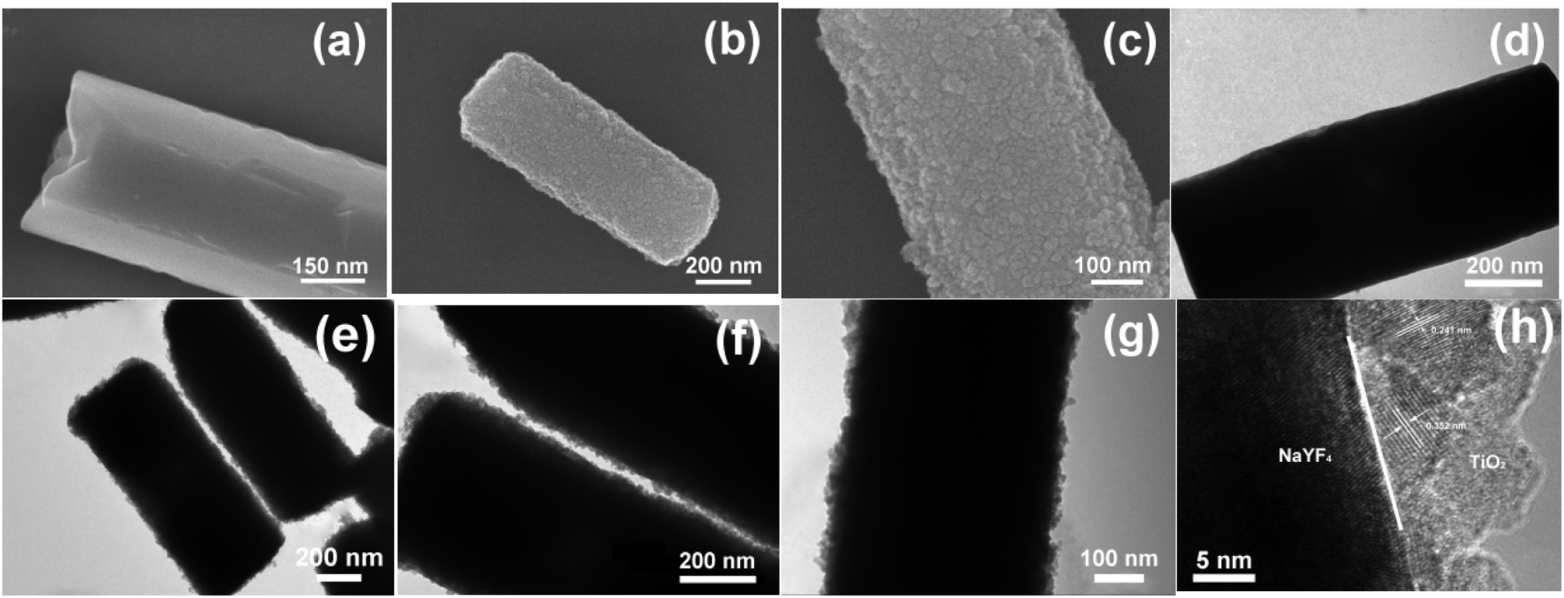
SEM and TEM images of pristine NaYF_4_:Yb^3+^,Tm^3+^ microcrystals (a and d) and TiO_2_-coated NaYF_4_:Yb^3+^,Tm^3+^ composites (b–c), (e–f). (h) is the HRTEM image of (g).

The morphology and size of the samples were analyzed by scanning electron microscopy (SEM) and transmission electron microscopy (TEM). Fig. 2 shows the SEM and TEM images of the NaYF_4_:Yb^3+^,Tm^3+^ microcrystals and the NaYF_4_:Yb^3+^,Tm^3+^@TiO_2_ photocatalysts. Fig. 2(a) and (d) show the SEM and TEM images of the NaYF_4_:Yb^3+^,Tm^3+^,Tm microcrystals, respectively. It can be observed that when EDTA was used as the surfactant and chelating agent, the samples obtained by the hydrothermal method were microrods. They were approximately 1.2 µm in length and about 0.4 µm in width, and their surface was relatively smooth and flat. However, after compositing with TiO_2_ particles, the surface of the microrods changed from smooth to rough, as shown in Fig. 2(b) and (e). Fig. 2(c) and (f) show the enlarged views of Fig. 2(b) and (e), respectively. It can be clearly seen from these figures that the shell layer composed of porous TiO_2_ nanoparticles was closely deposited on the surface of the microrods, and the thickness of the shell layer is approximately 10 nm. The size of a single TiO_2_ nanoparticle is about 6 nm. Fig. 2(g) shows the local TEM image of a single microrod, and Fig. 2(h) shows the corresponding high-resolution transmission electron microscopy (HRTEM) image. Through calculation, the interplanar spacings of the particles in the shell layer were found to be 0.352 nm and 0.24 nm, which are consistent with the interplanar spacings of the {100} and {103} crystal planes of the anatase structure of TiO_2_, respectively, confirming that the TiO_2_ on the surface of the microrods has an anatase structure. Within the field of view, all the TiO_2_ nanoparticles were deposited on the surface of the microrods, and there was no phenomenon of secondary nucleation. Repeated experiments have demonstrated that the two-step wet chemical method we adopted for synthesizing the core–shell structured materials is very simple, effective, and easy to repeat.

**Fig. 3:**
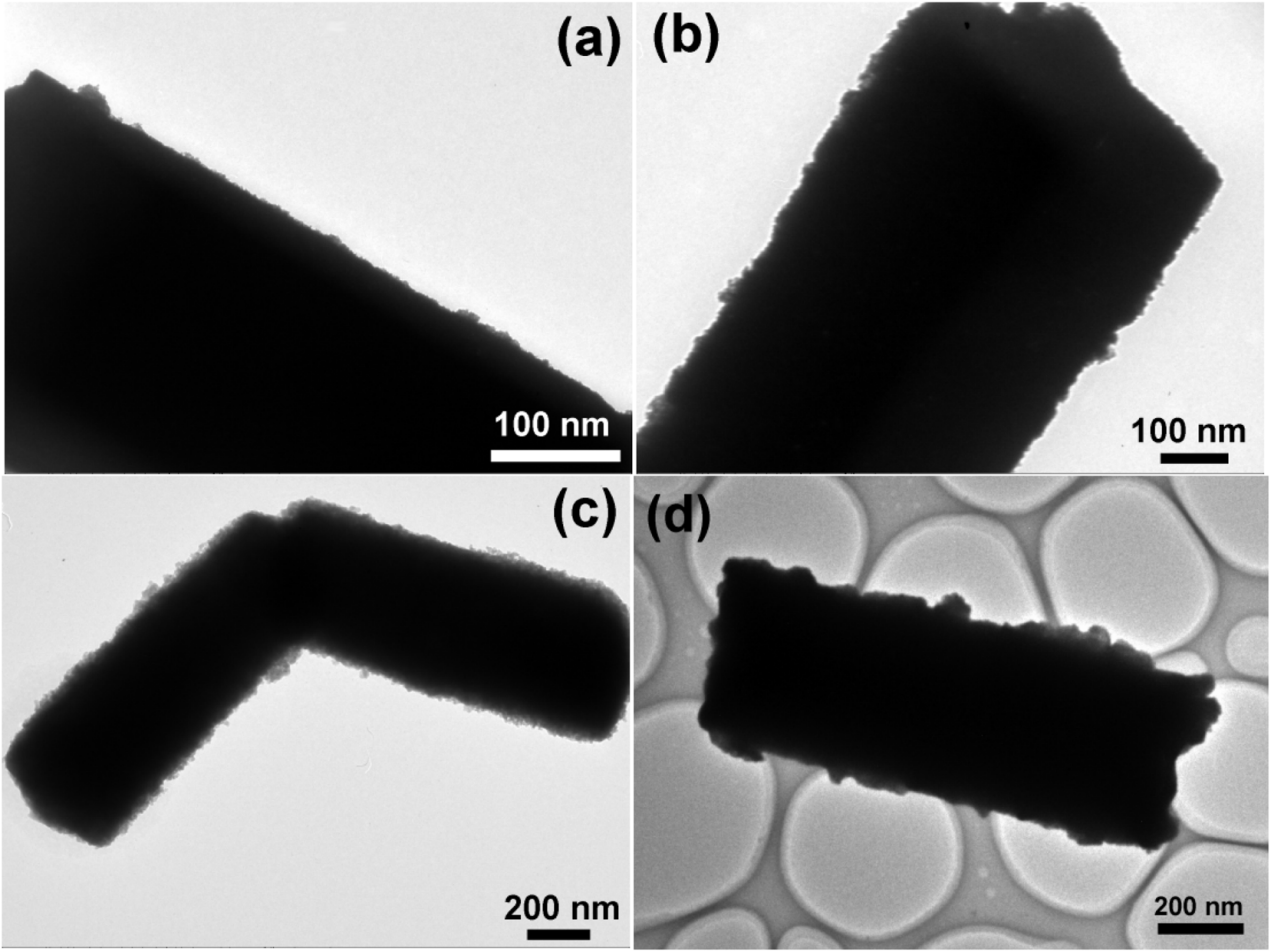
TEM images of S1 (a), S2 (b), S3 (c) and S4 (d).

Fig. 3 shows the TEM images of samples S1–S4. When a small amount of TBOT is added to the reaction precursor, a sparse TiO_2_ coating layer is formed on the surface of the micrometer crystals, as shown in Fig. 3(a). When the amount of TBOT in the reaction system is increased, a uniform and dense TiO_2_ coating layer is formed on the surface of the micrometer-sized crystals (Fig. 3(b)). When the amount of TBOT is further increased, the TiO_2_ shell layer is relatively uniform, and its thickness increases to about 15 nm (Fig. 3(c)). When the mass fraction of TiO_2_ in the reaction system is further increased, some of the TiO_2_ nanoparticles in the shell layer of the obtained composite material aggregate on the surface of the micrometer-sized rods (Fig. 3(d)).

### 3.2 Discussion of the optical properties

#### 3.2.1 Upconversion luminescence properties

**Fig. 4:**
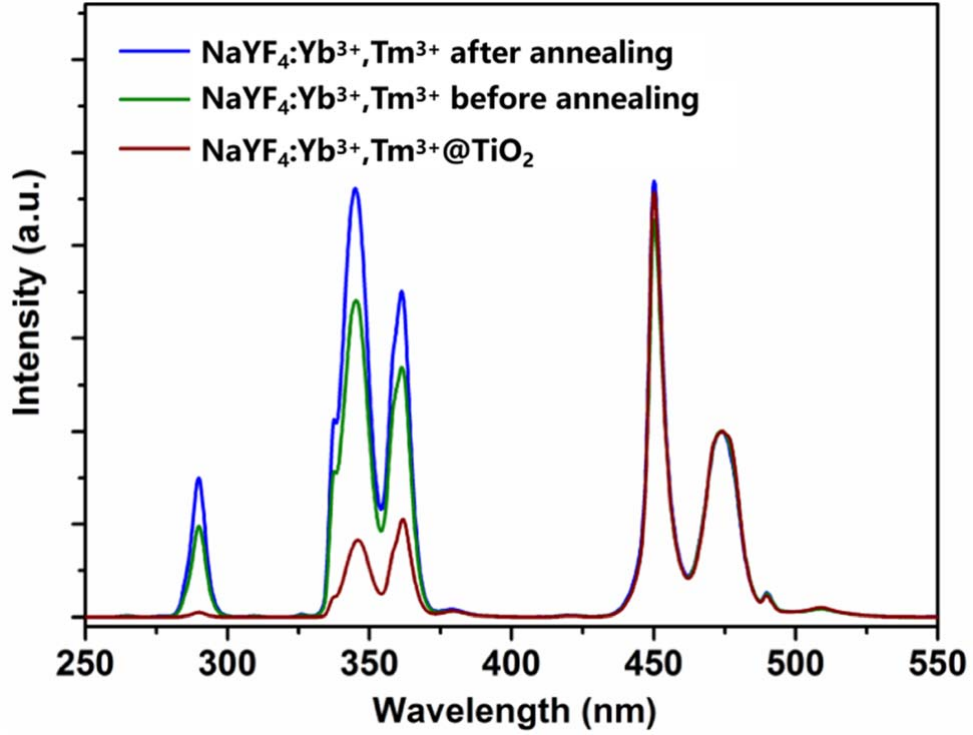
Upconversion emission spectra of the NaYF_4_:Yb^3+^,Tm^3+^ micrometer crystals before and after hydrothermal treatment and of the NaYF_4_:Yb^3+^,Tm^3+^@TiO_2_ composite material under 980 nm excitation.

The upconversion emission spectra of the samples were measured using a Hitachi F-4500 fluorescence spectrometer, with a 980 nm near-infrared semiconductor diode as the excitation source (excitation power: 100 mW, slit width: 1.0 nm, and PMT voltage: 400 V). Fig. 4 shows the upconversion fluorescence emission spectra of the NaYF_4_:Yb^3+^,Tm^3+^ microcrystals and the NaYF_4_:Yb^3+^,Tm^3+^@TiO_2_ composite materials. All the samples were normalized at the wavelength of 474 nm (^1^G_4_ → ^3^H_6_) to ensure the comparability of the luminescence intensity.

Fig. 4 shows the upconversion fluorescence emission spectra of the NaYF_4_:Yb^3+^,Tm^3+^ microrods before and after hydrothermal treatment, and of the NaYF_4_:Yb^3+^,Tm^3+^@TiO_2_ composite material. The emission peaks all arise from the characteristic emissions of Tm^3+^ ions. The ultraviolet emission peaks at wavelengths of 291 nm, 350 nm, and 365 nm result from the ^1^I_6_ → ^3^H_6_, ^1^I_6_ → ^3^F_4_, and ^1^D_2_ → ^3^H_6_ radiative transitions of Tm^3+^ ions, respectively. The two blue-light emission peaks at 450 nm and 474 nm are ascribed to the ^1^D_2_ → ^3^F_4_ and ^1^G_4_ → ^3^H_6_ radiative transitions of Tm^3+^ ions. By observing the upconversion emission spectra, it can be seen that the ultraviolet upconversion luminescence efficiency of NaYF_4_:Yb^3+^,Tm^3+^ significantly increased after annealing. This is mainly due to the presence of surface organic groups with high vibrational-mode energies, such as –OH and –NH_2_, on the surface of the unmodified particles before hydrothermal treatment. These organic groups and surface defects can easily cause non-radiative transitions of the excited-state energy levels of RE ions, leading to the quenching of the luminescent centers. Hydrothermal treatment can effectively remove a large number of surface states, such as organic groups and surface defects, reducing the probability of non-radiative transitions in the excited state and protecting the luminescent centers. Therefore, the ultraviolet-visible upconversion luminescence efficiency of the hydrothermal-treated NaYF_4_:Yb^3+^,Tm^3+^ microrods is enhanced.

However, when comparing the upconversion fluorescence emission spectra of the micro-rods before and after being coated with TiO_2_, it is observed that under near-infrared excitation, the intensities of the ultraviolet upconversion emissions at 291 nm, 350 nm, and 365 nm with wavelengths less than 400 nm are significantly weakened. Especially, the intensity of the emission peak at 291 nm (^1^I_6_ → ^3^H_6_) is very weak, while the fluorescence intensities of the two blue-light emission peaks (at 450 nm and 474 nm) hardly change. This phenomenon indicates that there are physical interactions such as transfer between NaYF_4_:Yb^3+^,Tm^3+^ and TiO_2_, rather than simple surface modification of TiO_2_ on the upconversion luminescent particles.

**Fig. 5:**
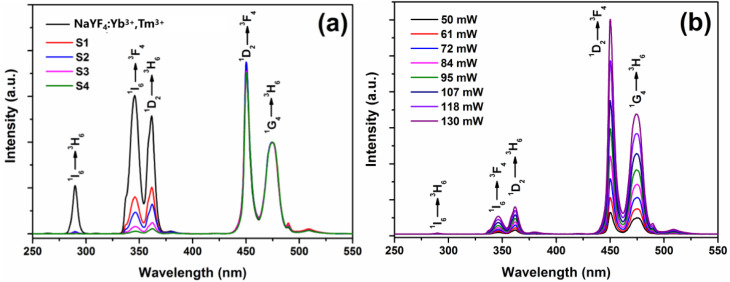
(a) Upconversion luminescence spectra of the NaYF_4_:Yb^3+^,Tm^3+^ micrometer-sized crystals and samples S1–S4 under 980 nm laser excitation. (b) Upconversion spectra of the sample S3 under different excitation powers.

Fig. 5(a) shows the upconversion luminescence spectra of NaYF_4_:Yb^3+^,Tm^3+^ microrods and sample S1 to S4 under the excitation of a 980 nm laser. Compared with the upconversion emission spectrum of NaYF_4_:Yb^3+^,Tm^3+^ microrods, the intensities of the upconversion emission peaks of samples S1–S4 in the blue-light region do not change significantly. However, in the ultraviolet band, the upconversion intensity decreases as the mass fraction of the TiO_2_ shell layer increase. This phenomenon can be attributed to two reasons: firstly, as the mass fraction of TiO_2_ in the composite material increases, the probability of re-absorption of upconverted ultraviolet light by the TiO_2_ shell layer effectively increases. Secondly, the increase in the mass fraction of TiO_2_ leads to an increase in the probability of energy transfer from Tm^3+^ ions in the ^1^I_6_ and ^1^D_2_ excited-state energy levels to the TiO_2_ shell layer in the composite material. Fig. 5(b) shows the upconversion luminescence spectra of sample S3 under different excitation light powers (50–130 mW). The intensities of all characteristic emission peaks of Tm^3+^ ions increase with the increase of the laser power, while the luminescence intensity at 291 nm (^1^I_6_ → ^3^H_6_) is still very weak. By analyzing Fig. 5(a) and (b) together, we conclude that the successive weakening of the ultraviolet upconversion luminescence intensities of the composite materials S1–S4 in Fig. 5(a) is caused by the increase in the mass fraction of TiO_2_, and has nothing to do with the intensity of the excitation light power.

#### 3.2.2 Absorption spectra

**Fig. 6:**
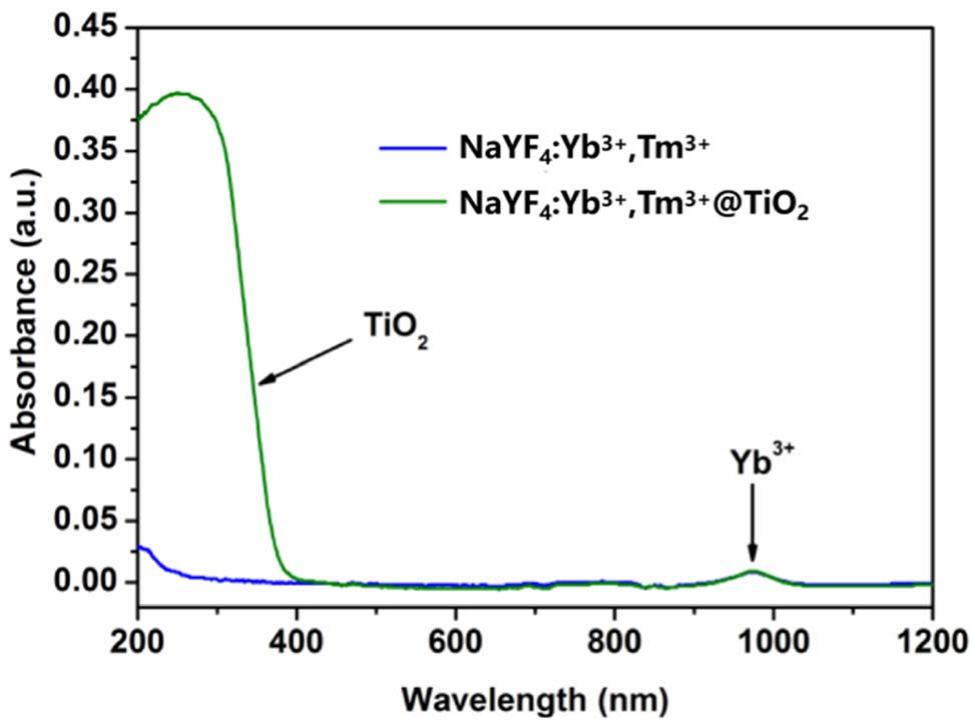
Absorption spectra of the NaYF_4_:Yb^3+^,Tm^3+^ micrometer-sized crystals and NaYF_4_:Yb^3+^,Tm^3+^@TiO_2_ composite materials.

The absorption spectra of the samples were measured using a Shimadzu UV-3600 UV-vis-NIR spectrophotometer in a scanning range of ∼200–1200 nm at a scanning rate of 200 nm min^−1^. The test results are shown in Fig. 6. The transition of Yb^3+^ ions from ^2^F_5/2_ → ^2^F_7/2_ gives the samples a relatively large absorption cross-section near 980 nm. Therefore, the absorption peaks near 980 nm in the absorption spectra of NaYF_4_:Yb^3+^,Tm^3+^ and NaYF_4_:Yb^3+^,Tm^3+^@TiO_2_ are the characteristic absorption peaks of Yb^3+^ ions. In the absorption spectrum of the NaYF_4_:Yb^3+^,Tm^3+^@TiO_2_ composite material, in addition to the characteristic absorption peak of Yb^3+^ ions, there is a very strong absorption band near a wavelength of 400 nm, which is the characteristic absorption band of anatase-structured TiO_2_. This combined with the phenomenon that the presence of TiO_2_ in the upconversion spectrum significantly weakens the ultraviolet emission strongly illustrates that the weakening of the upconversion ultraviolet luminescence intensity is partly caused by radiation absorption and reabsorption in the TiO_2_ shell layer.

#### 3.2.3 Fluorescence decay curve

The energy migration process between upconversion and TiO_2_ in the NaYF_4_:Yb^3+^,Tm^3+^@TiO_2_ microcomposite material was analyzed through the fluorescence decay curves of the three excited-state energy levels of ^1^I_6_, ^1^D_2_ and ^1^G_4_ in Tm^3+^ ions. The fluorescence decay curves were measured using a Hitachi F-4500 fluorescence spectrometer at an excitation wavelength of *λ*_ex_ = 953.6 nm and a time resolution of 0.1 µs.

**Fig. 7:**
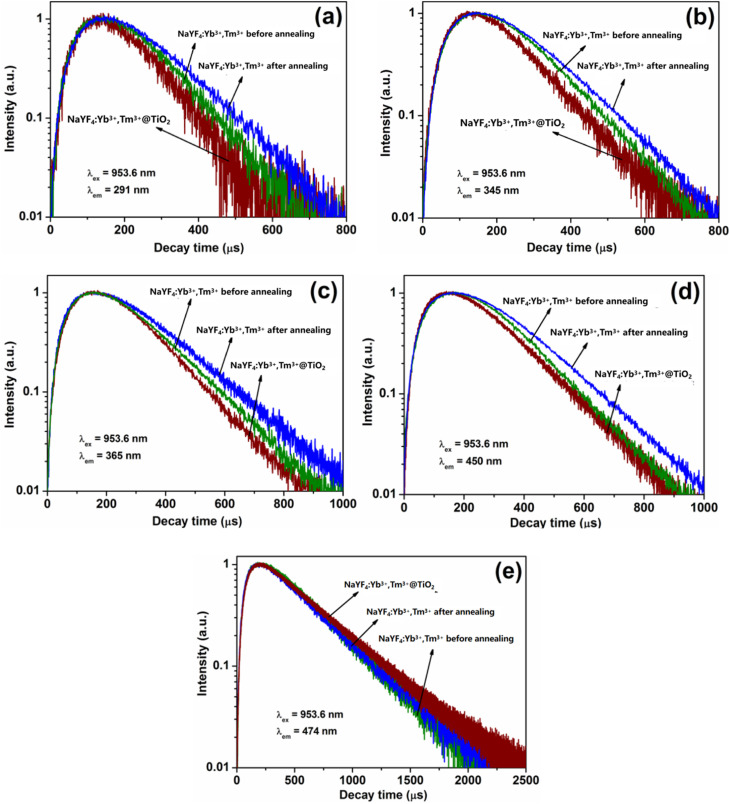
Fluorescence decay curves of the three excited-state energy levels of ^1^I_6_, ^1^D_2_ and ^1^G_4_ of Tm^3+^ ions in the NaYF_4_:Yb^3+^,Tm^3+^ microcrystals before (green) and after (blue) hydrothermal treatment and in the NaYF_4_:Yb,Tm/TiO_2_ composite material (red). The detected peaks at (a) 291, (b) 345, (c) 365, (d) 450 and (e) 474 nm correspond to the radiative transitions of ^1^I_6_ → ^3^H_6_, ^1^I_6_ → ^3^F_4_, ^1^D_2_ → ^3^H_6_, ^1^D_2_ → ^3^F_4_ and ^1^G_4_ → ^3^H_6_, respectively, and the excitation wavelength (*λ*) is 953.6 nm.

Fig. 7 shows the fluorescence decay curves obtained at different emission peaks measured for the three excited states of Tm^3+^ ions in the NaYF_4_:Yb^3+^,Tm^3+^ microcrystals before (green) and after (blue) hydrothermal treatment and in the NaYF_4_:Yb^3+^,Tm^3+^@TiO_2_ composite material (red), respectively at the excitation wavelength *λ* = 953.6 nm. The detected emission peaks are located at 291, 345, 365, 450 and 474 nm, corresponding to the radiative transitions of ^1^I_6_ → ^3^H_6_, ^1^I_6_ → ^3^F_4_, ^1^D_2_ → ^3^H_6_, ^1^D_2_ → ^3^F_4_ and ^1^G_4_ → ^3^H_6_, respectively. Each fluorescence curve is composed of two parts: a rising edge and a falling edge. In the rising edge part, the fluorescence intensity rapidly increases to the maximum value, indicating that the energy transfer process from Yb^3+^ ions to Tm^3+^ ions is rapid and effective. In the falling part, as the ultraviolet-visible light radiative transition of the excited-state energy levels in Tm^3+^ ions occurs, the fluorescence intensity decays in a single-exponential function due to the ultraviolet-visible light radiative transition of the excited-state energy levels in Tm^3+^ ions.

The decay part was fitted with a single-exponential decay formula, and the fitting values of the fluorescence lifetime are shown in Table 2. For the detected peak at 291 nm (corresponding to the ^1^I_6_ → ^3^H_6_ radiative transition), the energy level lifetime of the ^1^I_6_ excited state increases by approximately 16% (from 110 µs to 128 µs). This is because the reduction of surface defects and organic groups on the surface of the microrods after hydrothermal treatment leads to a decrease in the non-radiative transition rate (W_NIR), thus increasing the fluorescence lifetime of the excited state. Compared with NaYF_4_:Yb,Tm after hydrothermal treatment, the fluorescence decay lifetime of the ^1^G_4_ energy level of Tm^3+^ ions in the NaYF_4_:Yb^3+^,Tm^3+^@TiO_2_ composite system increases by about 10% (from 413 µs to 456 µs), which is mainly because the TiO_2_ shell layer reduces the surface defects in the NaYF_4_ particles and decreases the probability of non-radiative transition of the ^1^G_4_ energy level. In contrast, the fluorescence lifetime of the ^1^I_6_ energy level decreases by about 21% (from 128 µs to 100 µs). This result confirms that there is an effective energy transfer process between the upconversion microrods and the TiO_2_ shell layer in the composite system. The energies of the ^1^I_6_ and ^1^D_2_ excited state energy level are greater than the bandgap width of TiO_2_, and thus the Tm^3+^ ions in these two energy levels directly transfer energy to TiO_2_, leading to the accelerated decay of the fluorescence lifetime, while the energy of the ^1^G_4_ energy level is less than the bandgap width of TiO_2_ and cannot transfer energy to TiO_2_, and thus its fluorescence lifetime is prolonged due to the reduction of surface defects.

1.1 *I*_(*t*)_ = *A*_1_ exp(−*t*/*τ*_1_) + *A*_2_ exp(−*t*/*τ*_2_)

**Table 2:** Fluorescence lifetime of the ions at different excited states in different samples (µs)

Sample	^1^I_6_ (µs)		^1^D_2_ (µs)		^1^G_4_ (µs)
	291 nm	345 nm	365 nm	450 nm	474 nm
NaYF_4_:Yb^3+^,Tm^3+^ (before annealing)	110	115	150	148	387
NaYF_4_:Yb^3+^,Tm^3+^ (after annealing)	128	127	168	164	413
NaYF_4_:Yb^3+^,Tm^3+^@TiO_2_	100	112	134	142	456

The decay part is fitted with a formula, and the fitting values are shown in Table 2.

According to formula (1.1), the reduction of surface defects and organic groups on the surface of the microparticles after hydrothermal treatment leads to a decrease in the non-radiative transition rate (WNR), thus increasing the fluorescence lifetime of the excited state.

### 3.3 Standardized photocatalytic degradation experiments of methyl orange

#### 3.3.1 Experimental setup and operation parameters

All experiments for the photocatalytic degradation of methyl orange (MO) were carried out in a customized temperature-controlled photocatalytic reaction system to ensure the reproducibility of the test results. The key experimental parameters and detailed operation steps are clearly specified as follows, in accordance with the reproducibility requirements for photocatalytic experiments.

The pH value of the solution has a significant impact on the photocatalytic degradation rate. For different reaction systems, the influence of the pH value on the catalytic efficiency also varies. Herein, the organic dye system of methyl orange (MO) was taken as the research object to study the photocatalytic activity of the NaYF_4_:Yb^3+^,Tm^3+^@TiO_2_ microcomposite material in aqueous solutions with different pH values.

**Fig. 8:**
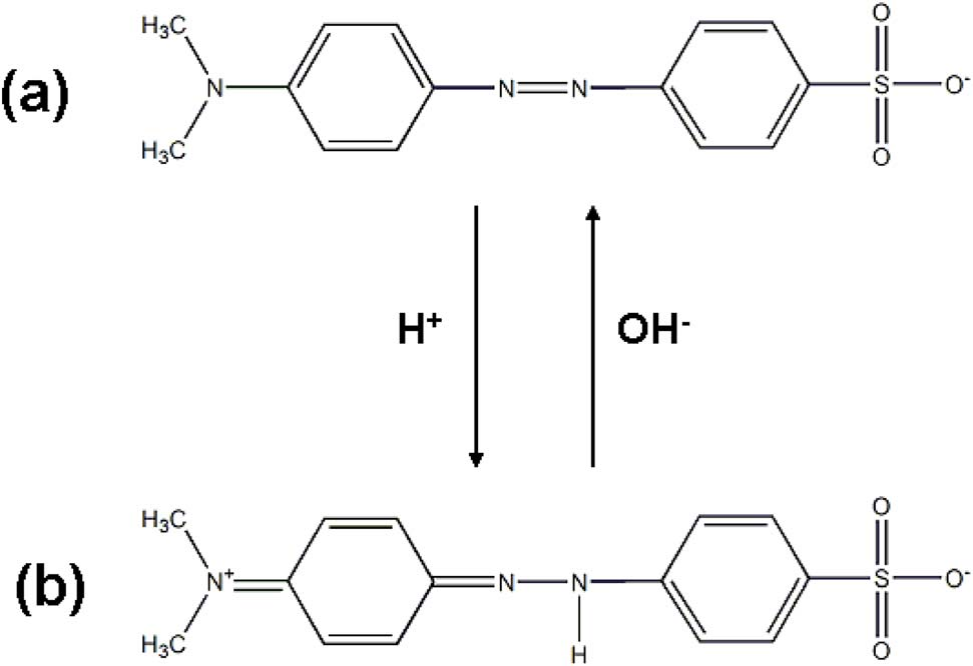
Molecular structure of MO in (a) neutral (orange-colored) and alkaline (yellow-colored) aqueous solutions featuring a typical azo structure. (b) Molecular structure of MO in an acidic (red-colored) aqueous solution, with a typical quinoid structure.

MO is an azo organic dye compound that is relatively difficult to degrade. Its molecule has two main structural forms: the azo structure (Fig. 8(a)) and the quinoid structure (Fig. 8(b)). Under alkaline and neutral conditions, the MO aqueous solution appears yellow and orange, respectively, and at this time, the MO molecules are all in the azo structure; under acidic conditions, the solution appears red, and MO is in the quinoid structure. Therefore, it is representative to study MO as a model dye compound. Due to the different molecular structures, the characteristic absorption peaks of MO in acidic and neutral (or alkaline) solutions are located at wavelengths of 506 nm and 464 nm, respectively. Here, under the conditions of an equal amount of photocatalyst, the same light irradiation time and light source intensity, the photocatalytic activity of the composite material was studied at pH values of 2, 6.5 and 11. In the experiment, the pH value of the MO aqueous solution was adjusted using 0.1 M HCl and 0.1 M NaOH aqueous solutions. The concentration of the photocatalyst was 1 mg mL^−1^. The excitation power density was 70 W cm^−2^.

#### 3.3.2 Photocatalytic degradation results and analysis

**Fig. 9:**
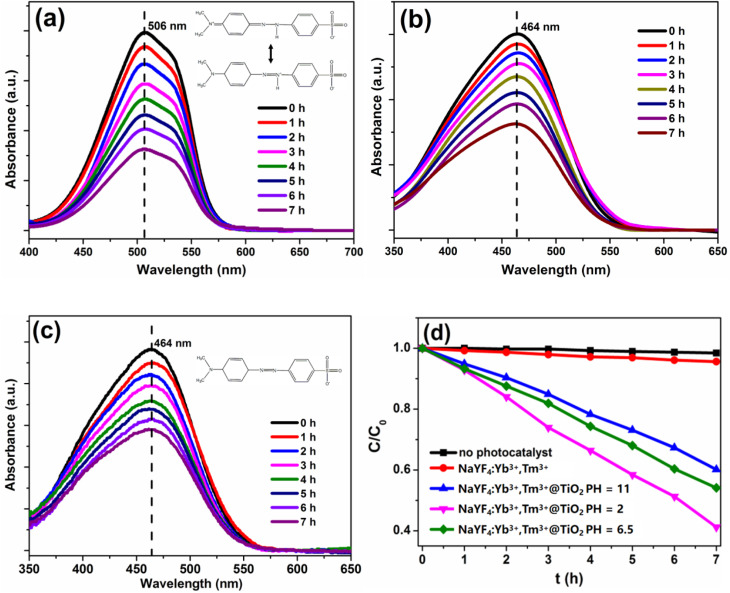
Change in the absorption spectrum of MO with the illumination time when the NaYF_4_:Yb^3+^,Tm^3+^@TiO_2_ photocatalyst is excited under NIR illumination in aqueous solutions of different pH values: (a) pH = 2, (b) pH = 6.5, and (c) pH = 11. (d) Change in the concentration ratio (*C*/*C*_0_) of methyl orange with an increase in the illumination time under different conditions: catalyst without illumination, illumination without catalyst, and illumination in the presence of the NaYF_4_:Yb^3+^,Tm^3+^@TiO_2_ composite material at different pH values.

Fig. 9(a)–(c) show the change in the absorption spectra of MO with illumination time in aqueous solutions of different pH values when NaYF_4_:Yb^3+^,Tm^3+^@TiO_2_ is excited by NIR. It can be seen that the characteristic absorption peak intensity of MO decreases gradually with the extension of NIR irradiation time in all three pH systems, indicating that the composite catalyst has NIR-driven photocatalytic degradation performance for MO in different acid-base environments^12^. The absorption peak intensity of MO in the acidic system (pH = 2) decreases the fastest, followed by the neutral system (pH = 6.5), and the slowest in the alkaline system (pH = 11), indicating that the solution pH has significant impact on the photocatalytic degradation efficiency of the composite catalyst.

Fig. 9(d) shows the changes in curves of the ratio of the concentration of MO to the initial concentration (*C*/*C*_0_) with an increase in illumination time under different experimental conditions (no catalyst, only NaYF_4_:Yb^3+^,Tm^3+^ microrods, NaYF_4_:Yb^3+^,Tm^3+^@TiO_2_ composite at pH = 2, 6.5 and 11). The experimental results show that: (1) without the catalyst, the MO concentration has almost no change under NIR irradiation for 7 h, indicating that MO has no photolysis under 980 nm NIR light; (2) the pure NaYF_4_:Yb^3+^,Tm^3+^ microrods have almost no photocatalytic degradation effect on MO, with a decolorization efficiency of only about 5% after 7 h of irradiation, indicating that the upconversion material itself cannot degrade MO; and (3) the NaYF_4_:Yb^3+^,Tm^3+^@TiO_2_ composite catalyst exhibits a significant photocatalytic degradation performance, and the decolorization efficiency is highly dependent on the solution pH: the decolorization efficiency reaches 98.2% at pH = 2, 76.5% at pH = 6.5, and only 52.3% at pH = 11 after 7 h of NIR irradiation, which is consistent with the change trend of the absorption spectra (Fig. 9).

For the MO organic dye system, the pH-dependent photocatalytic performance is mainly determined by two factors: on the one hand, the MO molecule exists in a quinoid structure under acidic conditions (pH = 2), which is more easily degraded by hydroxyl radicals (˙OH) than the azo structure in neutral/alkaline conditions. On the other hand, TiO_2_ is an amphoteric oxide, and its surface charge changes with the solution pH. Under acidic conditions, the TiO_2_ surface adsorbs H^+^ and becomes positively charged (TiOH_2_^+^), which can effectively adsorb the negatively charged MO molecules through electrostatic attraction, increasing the contact probability between MO molecules and the active species (˙OH) generated on the catalyst surfaces. Under alkaline conditions, the TiO_2_ surface adsorbs OH^−^ and becomes negatively charged (TiO^−^), which produces Coulomb repulsion with the negatively charged MO molecules, reducing the adsorption amount of MO and thus decreasing the photocatalytic degradation efficiency. The surface charge of TiO_2_ under neutral conditions is close to zero, and thus, the optimal photocatalytic performance is observed between acidic and alkaline conditions.

1.2 TiOH + H^+^ ↔ TiOH_2_^+^

1.3 TiOH + OH^−^ ↔ TiO^−^ + H_2_O

### 3.4 Post-reaction stability analysis

To evaluate the structural durability and reusability potential of the NaYF_4_:Yb^3+^,Tm^3+^@TiO_2_ core–shell photocatalysts in practical applications, the phase structure and surface chemical state of the S3 sample (with optimal photocatalytic performance) were characterized by X-ray diffraction (XRD) after the 12 h NIR-driven photocatalytic degradation of methyl orange (MO, pH = 2). The post-reaction catalyst was collected by centrifugation, washed thoroughly with deionized water and anhydrous ethanol to remove adsorbed MO molecules and reaction byproducts, and then dried at 80 °C in air prior to characterization, ensuring that no external impurities interfered with the test results.

**Fig. 10:**
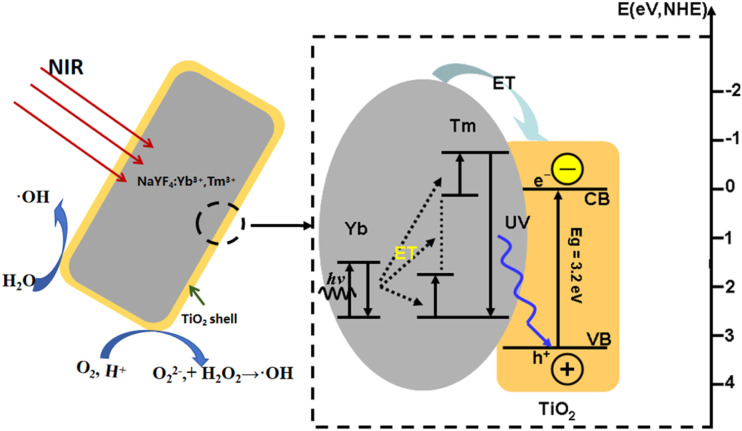
Schematic of the energy transfer process among Yb^3+^, Tm^3+^ and TiO_2_, the reaction of photogenerated electrons and holes with organic molecules on the TiO_2_ surface to generate ˙OH radicals, and the energy level diagram under near-infrared (NIR) light excitation.

Fig. 10 shows the XRD patterns of the NaYF_4_:Yb^3+^,Tm^3+^ and S3 samples after the photocatalytic reaction, with the standard reference patterns of hexagonal NaYF_4_ (JCPDS No. 16-0334) and anatase TiO_2_ (JCPDS No. 21-1272) overlaid for comparison. It can be observed that the XRD pattern of the post-reaction S3 sample is highly consistent with that of the fresh sample, where all characteristic diffraction peaks of hexagonal NaYF_4_ and anatase TiO_2_ remain well-resolved, with no obvious shifts in peak position, no significant reduction in peak intensity, and no new impurity diffraction peaks generated. The sharp diffraction peaks of NaYF_4_ and the weak characteristic peak of anatase TiO_2_ at 2*θ* = 25.4° are still clearly distinguishable, indicating that the core–shell crystal phase structure of NaYF_4_:Yb^3+^,Tm^3+^@TiO_2_ remains intact after the photocatalytic reaction. The high crystallinity of the composite effectively resists the structural damage caused by photochemical reactions and the acidic reaction environment (pH = 2), verifying the excellent phase stability of the photocatalyst during the degradation process.

### 3.5 Five-cycle reusability test

To evaluate the practical applicability and long-term performance stability of the NaYF_4_:Yb^3+^,Tm^3+^@TiO_2_ core–shell photocatalyst in actual wastewater treatment, five-cycle reusability tests were conducted under the optimal photocatalytic conditions (pH = 2, 50 mg catalyst, 50 mL 10 mg L^−1^ MO, at room temperature, 500 rpm magnetic stirring, 30 min dark adsorption, and 980 nm NIR with 7 W cm^−2^ power density), in accordance with the protocol for advanced oxidation catalysts in published work (DOI: 10.1039/D4NR02819H). The S3 sample with the optimal photocatalytic performance was selected as the test object, and the standard operation procedure for each cycle was strictly unified to ensure the comparability of the test results.

#### 3.5.1 Experimental procedure for reusability tests

(1) First cycle reaction: the S3 catalyst was added to an MO solution (pH = 2) for NIR-driven photocatalytic degradation, and the MO decolorization efficiency was determined after 7 h of irradiation according to the method in Section 3.3.1.

(2) Catalyst recovery and regeneration: after each cycle of reaction, the catalyst was rapidly separated from the reaction system by high-speed centrifugation (8000 rpm, 10 min), then thoroughly washed with deionized water and anhydrous ethanol, alternately, 3 times to remove the MO molecules and reaction byproducts adsorbed on the catalyst surface, and finally dried in an air-drying oven at 80 °C to a constant weight to ensure complete removal of the surface impurities.

(3) Subsequent cycles: the regenerated catalyst was reintroduced into fresh MO solution to carry out the next round of photocatalytic degradation reaction under identical experimental conditions. The above steps were repeated for a total of five consecutive cycles, and the MO decolorization efficiency of each cycle was recorded and calculated.

#### 3.5.2 Results and analysis of five-cycle reusability tests

The MO decolorization efficiency of the S3 sample in five consecutive photocatalytic degradation cycles is shown in Fig. 12, and the key performance data are summarized in Table 3. It can be clearly observed from the test results that the NaYF_4_:Yb^3+^,Tm^3+^@TiO_2_ composite photocatalyst maintains excellent catalytic activity and stability during five consecutive cycles. The MO decolorization efficiency remains above 94% even after the 5th cycle, and the relative retention rate of catalytic activity is still as high as 95% compared with the 1st cycle, showing no significant decline in performance. The slight decrease in catalytic activity (about 4.2% in total after five cycles) is mainly attributed to the minor loss of catalyst powder during the centrifugation, washing and transfer processes, rather than the structural damage or deactivation of the catalyst itself. No obvious agglomeration or structural collapse of the catalyst was observed during the test, which is consistent with the excellent phase and surface chemical stability verified by the post-reaction XRD characterizations.

**Table 3:** MO decolorization efficiency of the S3 sample in five consecutive NIR photocatalytic cycles (7 h irradiation and pH = 2)

Cycle number	Decolorization efficiency	Relative retention rate of the catalytic activity (*vs.* 1st cycle)
1st	98.2%	100.0%
2nd	97.5%	99.3%
3rd	96.8%	98.6%
4th	95.4%	97.1%
5th	94.1%	95.8%

**Fig. 11:**
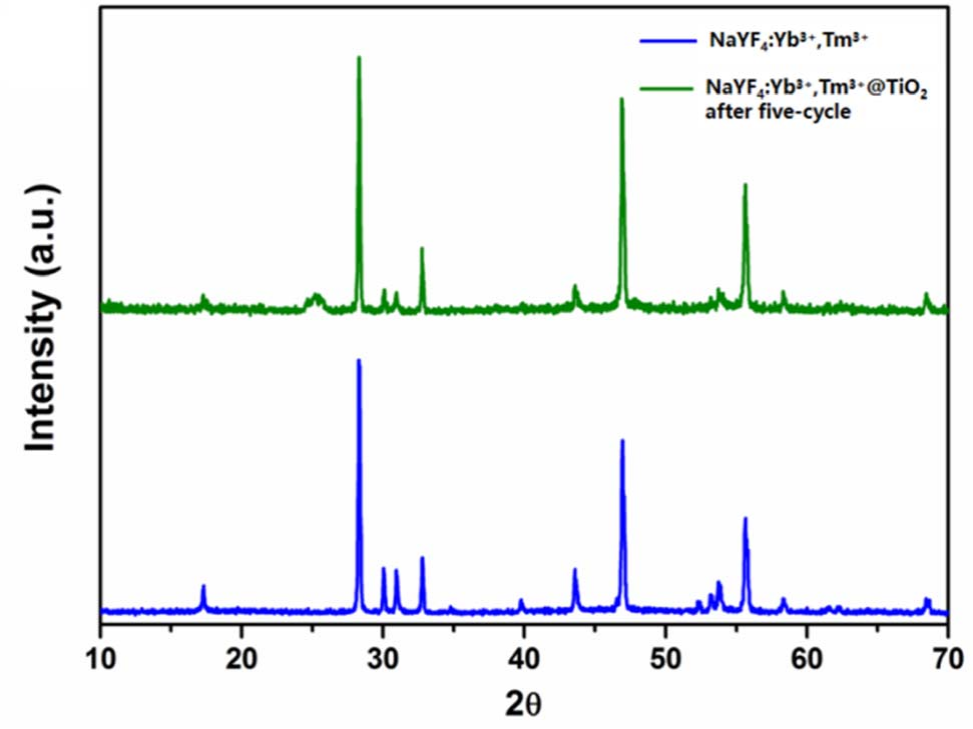
XRD patterns of the NaYF_4_:Yb^3+^,Tm^3+^ microrods and the S3 sample after five cycles of photocatalytic reaction. Note: fresh = before photocatalytic reaction and post-reaction = after 12 h of NIR-driven MO degradation (pH = 2), washed and dried.

**Fig. 12:**
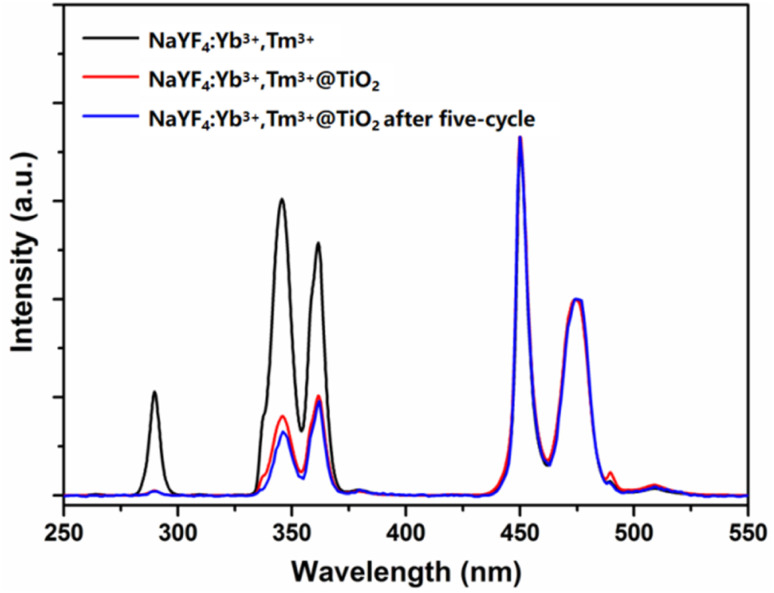
Upconversion luminescence spectra of the NaYF_4_:Yb^3+^,Tm^3+^ microrods and S3 sample after the 5th cycle compared with the fresh sample under a 980 nm laser excitation. Note: fresh = before photocatalytic reaction.

To further verify the structural durability of the catalyst after long-term cyclic use, Fig. 11 presents the upconversion luminescence (UCL) spectra of the fresh NaYF_4_:Yb^3+^,Tm^3+^ microrods, the fresh S3 sample, and the S3 sample after the 5th cycle, all measured under 980 nm laser excitation. Notably, all the characteristic upconversion emission peaks of Tm^3+^ ions (*e.g.*, 450 nm, 475 nm, and 650 nm) in the 5th-cycle S3 sample are well retained, with no significant shift in peak positions relative to the fresh sample. The relative intensities of these emission peaks also remain nearly unchanged, demonstrating that the upconversion luminescence performance of the NaYF_4_:Yb^3+^,Tm^3+^ core, which is critical for NIR light harvesting and efficient energy transfer to the TiO_2_ shell, is fully preserved after prolonged cyclic use. This result further confirms that the core–shell structure of the NaYF_4_:Yb^3+^,Tm^3+^@TiO_2_ composite remains structurally intact, and the optical properties essential for photocatalytic activity are stably maintained, providing robust evidence for the excellent long-term structural durability of the catalyst in repeated applications (Fig. 12).

## 4. Photocatalytic mechanism of NaYF_4_:Yb^3+^,Tm^3+^@TiO_2_ composites

**Fig. 13:**
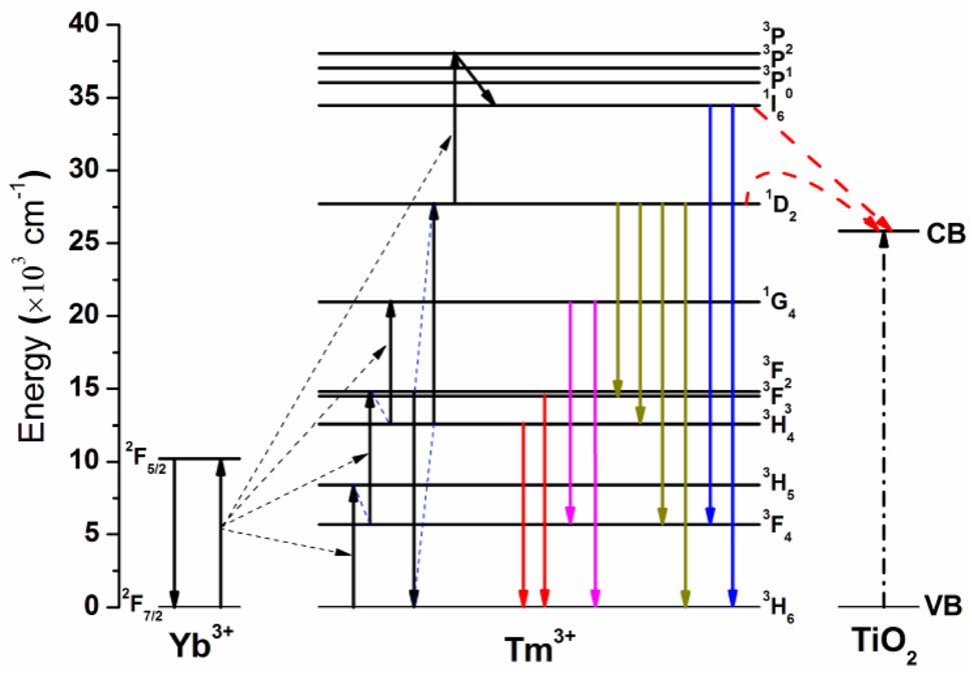
Energy level diagram of Yb^3+^–Tm^3+^ and the upconversion luminescence processes in an Yb^3+^–Tm^3+^ co-doped system under 980 nm excitation.

Fig. 13 illustrates the energy level diagrams of Yb^3+^–Tm^3+^ and the upconversion luminescence processes in an Yb^3+^–Tm^3+^ co-doped system upon 980 nm NIR excitation, as well as the energy transfer and photocatalytic degradation mechanism of the NaYF_4_:Yb^3+^,Tm^3+^@TiO_2_ composite catalyst. The 980 nm NIR pump light only excites the Yb^3+^ ions (^2^F_7/2_ → ^2^F_5/2_) and three successive energy transfers from Yb^3+^ to Tm^3+^ populate the ^3^H_5_, ^3^F_2_, and ^1^G_4_ levels. The ^1^D_2_ level of Tm^3+^ cannot be populated directly by the fourth photon from Yb^3+^*via* energy transfer due to the large energy mismatch (about 3500 cm^−1^) between them, and the cross-relaxation between Tm^3+^ ions is responsible for populating the ^1^D_2_ level. Usually, there are primarily two cross-relaxation processes that populate the ^1^D_2_ level: ^3^F_2_ + ^3^H_4_ → ^3^H_6_ + ^1^D_2_, and ^1^G_4_ +^3^H_4_ → ^3^F_4_ + ^1^D_2_. In addition, the ^1^D_2_ state can be promoted to the ^3^P_2_ state *via* another energy transfer from excited Yb^3+^ and then relaxes non-radiatively to the ^1^I_6_ level.

In the NaYF_4_:Yb^3+^,Tm^3+^@TiO_2_ composite system, the energies of the ^1^I_6_ and ^1^D_2_ levels of the Tm^3+^ ions are greater than the bandgap width of TiO_2_ (3.2 eV). The Tm^3+^ ions at these two energy levels (^1^I_6_ and ^1^D_2_) can directly and effectively transfer energy to the surrounding TiO_2_ through the fluorescence resonance energy transfer (FRET) process, which is the core mechanism for enhancing the NIR-driven photocatalytic activity of the composite catalyst. When TiO_2_ in the composite material is activated by the transferred energy, the electrons in the valence band (VB) transition to the conduction band (CB) and become photogenerated electrons (e^−^), leaving photogenerated holes (h^+^) in the valence band at the same time.

Part of the photogenerated electron–hole pairs recombine either inside the material or after migrating to the surface, releasing energy in the form of heat. Another part migrates from the interior of the semiconductor to its surface to participate in the corresponding redox reactions: the excited electrons that migrate to the surface of TiO_2_ react with the oxygen molecules (O_2_) or H^+^ adsorbed on its surface to generate O_2_^−^ or O_2_^2−^ and hydrogen peroxide (H_2_O_2_). H_2_O_2_ can react with superoxide anion radicals to produce hydroxyl radicals (˙OH). Meanwhile, the photogenerated holes react with H_2_O to generate hydroxyl radicals (˙OH). The generated hydroxyl radicals (˙OH) have strong oxidizing properties and are extremely reactive. The chemical reactions in which they participate proceed at a very rapid rate and belong to free radical reactions. ˙OH rapidly breaks the N–N and C–C bonds in the molecular structure of MO adsorbed on the surface of TiO_2_ particles, generating inorganic small molecular substances, thus achieving the purpose of degrading organic dye molecules.

In addition, the ^1^G_4_ energy level of Tm^3+^ ions has a lower energy than the bandgap width of TiO_2_, and thus no energy transfer occurs between this energy level and TiO_2_, which is consistent with the result that the blue-light upconversion luminescence intensity of the composite material remains almost unchanged after TiO_2_ coating.

## 5. Conclusion

This study provides a feasible preparation method for NaYF_4_:Yb^3+^,Tm^3+^-based upconversion@TiO_2_ core–shell composite photocatalysts and clearly clarifies their intrinsic energy transfer mechanism under NIR-driven photocatalytic performance. The as-prepared NaYF_4_:Yb^3+^,Tm^3+^@TiO_2_ microrod composites integrate excellent NIR photocatalytic activity, robust structural and chemical stability, and favorable reusability, which endows them with great practical application potential in solar-driven organic dye wastewater treatment. Beyond the material preparation and performance validation, this work also reveals the core role of fluorescence resonance energy transfer (FRET) between the NaYF_4_:Yb^3+^,Tm^3+^ upconversion core and anatase TiO_2_ shell in enhancing NIR photocatalysis, verifying that energy transfer from the Tm^3+^ excited states to TiO_2_ efficiently generates photogenerated electron–hole pairs and subsequent ˙OH radicals for pollutant degradation. The systematic investigation of pH-dependent photocatalytic performance also provides clear guidance for the practical application of the composite catalyst in actual wastewater systems with varying acid–base properties. In summary, this research not only develops a high-performance NIR-responsive photocatalyst for environmental remediation but also provides valuable theoretical and experimental insights for the rational design and fabrication of advanced upconversion-based composite photocatalysts, laying a solid foundation for the further development of solar light-driven photocatalytic technologies for water pollution control.

## Conflicts of interest

There are no conflicts to declare.

## Supplementary Material

RA-016-D6RA00088F-s001

## Data Availability

All data supporting the findings of this study, including structural, optical and photocatalytic data, are available in the article and Supplementary Information (SI). Supplementray information is available. See DOI: https://doi.org/10.1039/d6ra00088f.
