# Supplementary material for: Photocatalytic degradation of methyl orange at different pH values by NaYF4:Yb3+,Tm3+@TiO2 microrod composite photocatalysts under NIR excitation
Source: RSC Adv. 2026 May 29;16(32):29195–206. doi: 10.1039/d6ra00088f (PMC13223935; doi:10.1039/d6ra00088f)
Supplement: RA-016-D6RA00088F-s001 [file RA-016-D6RA00088F-s001.pdf]

Supplementary Information (SI) for RSC Advances.
This journal is © The Royal Society of Chemistry 2026

Supplementary Information

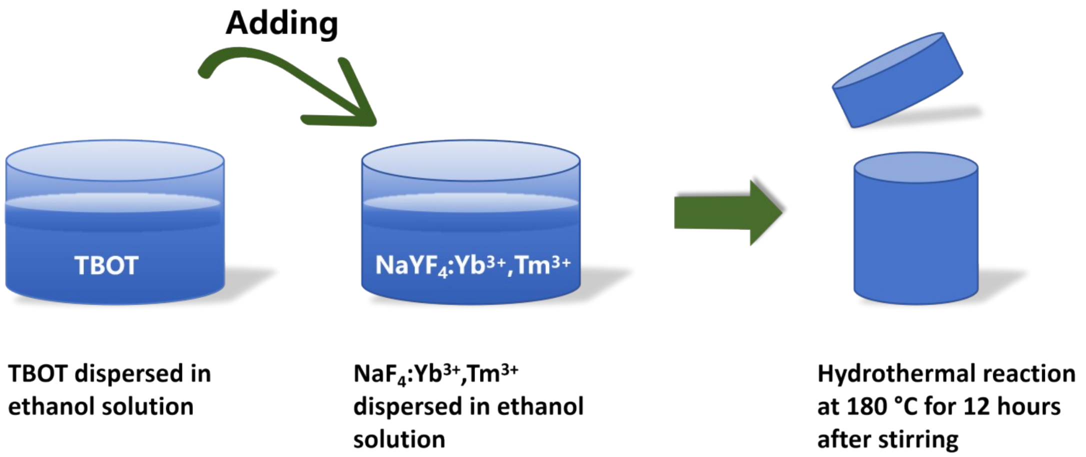

Figure Schematic synthesis route of the as-prepared $NaYF_4$:Yb^{3+},Tm^{3+}@TiO_2 microrod composite photocatalysts
